# Instruments for measuring self-management and self-care in geriatric patients – a scoping review

**DOI:** 10.3389/fpubh.2023.1284350

**Published:** 2023-12-15

**Authors:** Rebecca Wientzek, Rosa Marie Brückner, Aline Schönenberg, Tino Prell

**Affiliations:** ^1^Department of Geriatrics, Halle University Hospital, Halle (Saale), Germany; ^2^Department of Neurology, Jena University Hospital, Jena, Germany

**Keywords:** self-management, self-care, instrument, geriatric patients, geriatric syndromes

## Abstract

**Introduction:**

With demographic changes, prioritizing effective care for geriatric patients to maintain functionality, independence, and quality of life is crucial. Well-developed self-management or self-care abilities, which can be maintained and improved through interventions, are of the utmost importance. To implement these interventions tailored and effectively, a thorough assessment of the individual’s self-management and self-care abilities is required.

**Objective:**

This scoping review aimed to identify self-management and self-care instruments suitable for geriatric patients, their underlying theories and definitions of self-management and self-care, and their similarities and differences in item content.

**Methods:**

A systematic search of the PubMed and CINAHL databases was conducted to identify retrievable full-text articles published in English in the medical and nursing fields since the 1970s, which were validated on a sample with an average age of at least 70 years, used generic rather than disease-specific items, and addressed the broad range of self-management and self-care abilities.

**Results:**

Of the 20 included articles, six instruments were identified that were based on different theories and offered varying definitions of self-management or self-care. Despite these differences, all emphasize empowered individuals taking an active role in their care. Most address actual behavior and abilities referring to lifestyle factors and (anticipated) adjustment behavior. However, crucial aspects, such as psychological factors, (instrumental) activities of daily living, and social environment are not fully addressed in these instruments, nor are the types of execution to which the items refer, such as wants, feelings, confidence, or attitudes.

**Conclusion:**

To fully understand how geriatric patients implement daily self-management or self-care, a combination of instruments covering the important factors of self-management and self-care and addressing multiple types of item execution, such as behaviors, abilities, wants, or attitudes, is recommended. This review provides the first comprehensive overview of self-management and self-care instruments suitable for geriatric patients.

## 1 Introduction

Over the past 200 years, the average life expectancy has increased rapidly due to medical advances and improved living conditions and is expected to continue to rise (1). Aging successfully has, therefore, become a worldwide aim (2), often used synonymously to well-being (2) or adaptation to changes during the aging process (3). By 2050, the proportion of people aged 65 years or older in the world’s population is expected to increase from 9.1% in 2019 to 15.9% (4). Examining the prognosis explicitly for Europe and North America, it is expected that in 2050, one in four people will be over 65 years old. In the course of this development, the prevalence of chronic conditions and multiple chronic conditions, hence multimorbidity, is increasing, especially among older people (5–7). Those affected face challenges in managing their medications, symptoms, and disabilities as well as in coping with and adjusting to the changes associated with their health status and the accompanying psychosocial consequences (8–10). Among older adults, geriatric patients are particularly vulnerable. Geriatric patients are characterized by the presence of geriatric-typical multimorbidity (e.g., immobility, cognitive deficits, or incontinence) (11). Both health status and inadequate management of the challenges associated with health status can lead to deterioration of health and increased hospitalization (12–15). Notably, of all Germans hospitalized in 2021, the proportion of those over 65 years of age was approximately 44% (16). This is of particular concern because hospitalization itself is a risk factor for loss of functionality (17, 18) and frailty (3). Therefore, the geriatric treatment approach for older patients is resource-oriented and aims to maintain functionality, independence, and the associated quality of life (11). Self-management abilities play a crucial role in achieving these goals, which additionally minimize the burden on the healthcare system (15, 18–20).

### 1.1 Conceptual framework of self-management and self-care

Since the 1960s, self-management has been applied under the premise that patients actively participate in their treatment, and an integrated approach has been developed in which patients, families, and healthcare professionals are actively involved (21). Being able to successfully manage the physical (e.g., diet adherence), social (e.g., social contact), and psychological aspects (e.g., well-being or coping) of life is of utmost importance to age successfully despite health-related obstacles (2, 22). Self-management abilities have been associated with improved health distress (23, 24), self-efficacy (23, 24), and well-being (25–27). They further lead to a reduction in healthcare resource utilization (23, 28, 29). Although self-management has been widely researched, there is a lack of uniformity concerning the definition and concept of self-management, complicating the basic understanding and measurement of self-management (21, 30, 31). While some definitions focus on diagnosis-controlling behaviors (e.g., medication use or symptom management), others emphasize healthy lifestyle behaviors (e.g., exercise or diet) (19). The third category combines both types of behavior. For example, according to Corbin and Strauss (8), self-managing a chronic condition involves tasks in three domains: medical management of the condition, behavior management and emotional management. In these domains, six behaviors are executed: problem solving, decision making, resource utilization, forming a relationship with a healthcare provider, taking action, and self-tailoring (32, 33). Daily decisions about diet, exercise, and medication use reflect an individual’s self-management style (32, 34). It is therefore not possible, not to self-manage and the question of self-management is not about “if” but “how” (21).

Self-efficacy (21, 28, 31, 32), health literacy (21, 28, 31), social environment, monitoring behavior, and relationship with healthcare provider represent important antecedents for successful self-management, which therefore may predetermine the “how.” In accordance with Banduras social cognitive theory “perceived self-efficacy refers to beliefs in one’s capabilities to organize and execute the courses of actions required to produce given attainments” (p. 3) (35). Thus, self-efficacy serves as a predictive factor for adaptive behavior, which is crucial when confronted with illness (36, 37). Self-efficacy is also a consequence of self-management programs, as they primarily enhance self-efficacy, which influences health utilization via a direct effect on health status (38, 39).

A concept closely related to self-management, which is often used interchangeably (39, 40) and has a large overlap concerning theoretical background, outcomes, and determinants, is self-care. For example, self-efficacy (40, 41), social environment, health literacy (41), and monitoring behavior (40) are also important antecedents of self-care. Much research in this field is based on the theory of nursing scientist Dorothea Orem’s theory that self-care is “the practice of activities that individuals initiate on their own behalf in maintaining life, health and well-being” (p. 84) (42). These activities not only refer to activities of daily living but also to monitoring of symptoms, treatment, and medical adherence as well as social and psychological aspects (43, 44). The power of individuals to engage in those self-care activities is called self-care agency, therefore encompassing self-care ability (43). Self-care agency varies according to a person’s development and is determined by age, health status, cultural background, and life experiences (43). Particularly for older adults, self-care is a resource that helps them maintain their health and remain community-dwelling for as long as possible (45). Successful engagement in self-care is associated with improved quality of life (46, 47), reduced hospitalization, and mortality rates (14). Overall, however, clearly defining self-care and the factors that underpin it pose the same problem as defining self-management, leading to an incomplete assessment of self-care (41) and the aforementioned interchangeable use of the two concepts (40, 48). While some view self-management as a subset of self-care (48), others argue that self-care is subordinate to self-management (49). In fact, Riegel et al. (50) combined the two constructs under the term “self-care management” to refer to symptom management. Because of these blurred boundaries, self-management research should consider the current state of self-care research to get a full picture of how people manage their health.

### 1.2 Improving and assessing self-management and self-care abilities

In recent decades, numerous self-management and self-care interventions have been developed and evaluated in randomized controlled trials. Self-management interventions have often utilized social cognitive theory, such as the Stanford Chronic Disease Self-Management Program (CDSMP) (39, 51). These interventions have resulted in various health-related improvements across various conditions including arthritis (52, 53), heart failure (54), diabetes (24, 55) or asthma (56). However, most of those interventions are disease-specific, and therefore less suitable for geriatric patients, who suffer from a multitude of health problems that radiate into all areas of life and are sometimes self-reinforcing. Therefore, geriatric patients require comprehensive self-management interventions that address all of these areas.

To support patients effectively, it is necessary to assess their individual self-management abilities and resources so that interventions can be tailored to their unique needs (57). To date, a vast number of instruments have been developed to assess generic or disease-specific self-management and self-care abilities of all ages and in numerous contexts. Numerous reviews have been conducted with the aim to provide a comprehensive overview of said instruments, with varying degrees of focus. This includes reviews of instruments measuring specific self-management abilities (58), disease-specific self-management or self-care (59–63), generic self-management or self-care (30), and disease-specific or generic self-management or self-care (64, 65). None of these reviews focused on generic self-management or self-care instruments that are suitable for geriatric patients. Hence, we addressed these important issues in this review. Due to the aforementioned ambiguity regarding the definitions and concepts of self-management, a general comparability of the instruments is not possible (21, 30, 31). Therefore, a scoping review was performed instead of a systematic review (66).

### 1.3 Objectives

This scoping review aimed to capture the state of research in the medical and nursing fields on retrievable English-language instruments for assessing the broad range of self-management and self-care abilities in geriatric patients (≥ 70 years) using generic rather than disease-specific items. The authors’ definitions of self-management and self-care as well as the underlying concepts of the constructs were of keen interest. Thus, we sought to answer the following questions in the course of this scoping review: (I) What generic self-management or self-care instruments are suitable for geriatric patients? (II) Which underlying theories are these instruments based on? (III) How do the authors of these instruments define self-management or self-care? (IV) What commonalities and differences can be identified in the item contents of the instruments?

## 2 Method

The PRISMA Extension for Scoping Reviews (PRISMA-ScR) reporting guidelines (67) were followed for this scoping review.

### 2.1 Eligibility criteria

To be considered for our review, studies and instruments had to be validations or developments of a self-management or self-care instrument, which was validated on a sample with an average age of at least 70 years or older, use generic rather than disease-specific items, and address the overall concept of self-management and self-care with its broad range of abilities rather than specific aspects, such as adherence or self-efficacy. All English-language, full-text primary research articles published between 1970 and 2023 were included.

### 2.2 Information sources

A comprehensive literature search was conducted in April and October 2023 using the PubMed and CINAHL databases to identify relevant articles published in the medical and nursing fields. In addition, the original development studies of those instruments that met the eligibility criteria and were not found in the database search were identified by hand search.

### 2.3 Search strategy

For the data collection the following keywords were used and combined with the Boolean operators “OR” and “AND”: self-management, self-care, questionnaire, instrument, scale, validation, development, measure and assess. The search strategy was drafted by the authors and is provided in detail in the Supplementary Figure S1.

### 2.4 Selection of sources of evidence

A four-step process was used to extract results from the databases. First, results from both databases were exported as .csv files, and merged into one Excel file. This file was screened for duplicates and these were removed. The titles and abstracts of the remaining articles were then reviewed for compliance with the eligibility criteria, resulting in the exclusion of the majority of the articles. In the fourth step, the full texts of the remaining articles were screened and those that did not sufficiently meet the eligibility criteria were excluded.

### 2.5 Data charting process

To systematically extract and summarize relevant information from the included studies, we developed a comprehensive extraction form with two spreadsheets. While the first spreadsheet contained information about the theoretical background of the instruments and their characteristics, the second focused on the item content. The extraction form was stored on our internal server so that any team member could access, download, update, and upload it. We completed the first spreadsheet with verbatim excerpts from the eligible studies according to the variables agreed upon, and the remaining ambiguities were resolved through discussion. In the second spreadsheet, the items of all instruments were first entered and then examined for similarities and contrasts with respect to the relevant factors of self-management and self-care extracted from the literature. After an initial independent review, the results were compared and discrepancies were discussed and resolved.

### 2.6 Data items and synthesis of results

In the course of the data charting process, we extracted the following information in the first spreadsheet: name of the instrument and its abbreviation, authors involved, year of publication, definition of self-management or self-care, underlying theory on which the instrument was based, reason for development, number of items, number and names of scales and factors, psychometric results, and characteristics of the sample. Two tables were created to summarize the results for these variables. In the theoretical background and sample-focused Supplementary Table S1, the name of the instrument, the source of the original instrument and that of the adaptation of the selected validation study on people aged ≥70 years, the sample characteristics of the latter, and the underlying theory and definition are contrasted. A more detailed comparison of the instruments is shown in Supplementary Table S2, which contrasts the number of items, rating scales, identified factors and subscales, and internal consistency of the studies in which the instruments were tested and validated in the target population. Only view studies have described the reasons for their development. Therefore, this variable was not included in the tables.

In the second spreadsheet, the content of each item was compared to the following categories, and it was noted where they were applicable or not: psychological and emotional aspects, (instrumental) activities of daily living, social environment, (anticipated) disease controlling behavior, lifestyle factors, (anticipated) adjustment behavior, relationship with health care provider, monitoring of symptoms, treatment and medication (side effects), and health literacy. In addition, the items were checked for the type of item execution such as behavior, ability, knowledge/understanding, attitude, feelings, want to do something, and confidence of doing something. Based on these results, two tables were created. Table 1 shows which content categories are included in the individual items of the instruments, while Table 2 compares the types of execution the items of the instruments refer to. The reviewers added up the frequencies and marked the most frequently occurring content category and type execution per instrument accordingly.

**Table 1:** Item content of the generic instruments measuring self-management or self-care.

Item content	ASAS / ASAS-R	PAM-13	PIH-OA	SASE	SC-CII	SMAS-30
Psychological/Emotional aspects	X	X	X	X	X	X
(Instrumental) activities of daily living			X	**X**		
Social environment	X		X			**X**
(Anticipated) disease-controlling behavior	**X**	**X**	X		X	
Health literacy	X	X	**X**		X	
Lifestyle factors	X		X	X	X	X
(Anticipated) adjustment behavior	X	X	X	X	X	
Relationship with healthcare provider		X			X	
Monitoring behavior	X	X	X		**X**	

Bold X’s indicate the most common item content in the instrument.

**Table 2:** Types of item execution of the generic instruments measuring self-management or self-care.

Manifestation	ASAS / ASAS-R	PAM-13	PIH-OA	SASE	SC-CII	SMAS-30
Behavior	**X**	X	X	X	**X**	**X**
Abilities	X	X	**X**	**X**		X
Knowledge, understanding		X	X	X	X	
Attitude		X				X
Feelings				X		
Wants				X		
Confidence		**X**				X

Bold X’s indicate the most common types of item execution in the instrument.

## 3 Results

### 3.1 Selection of sources of evidence

**Figure 1:**
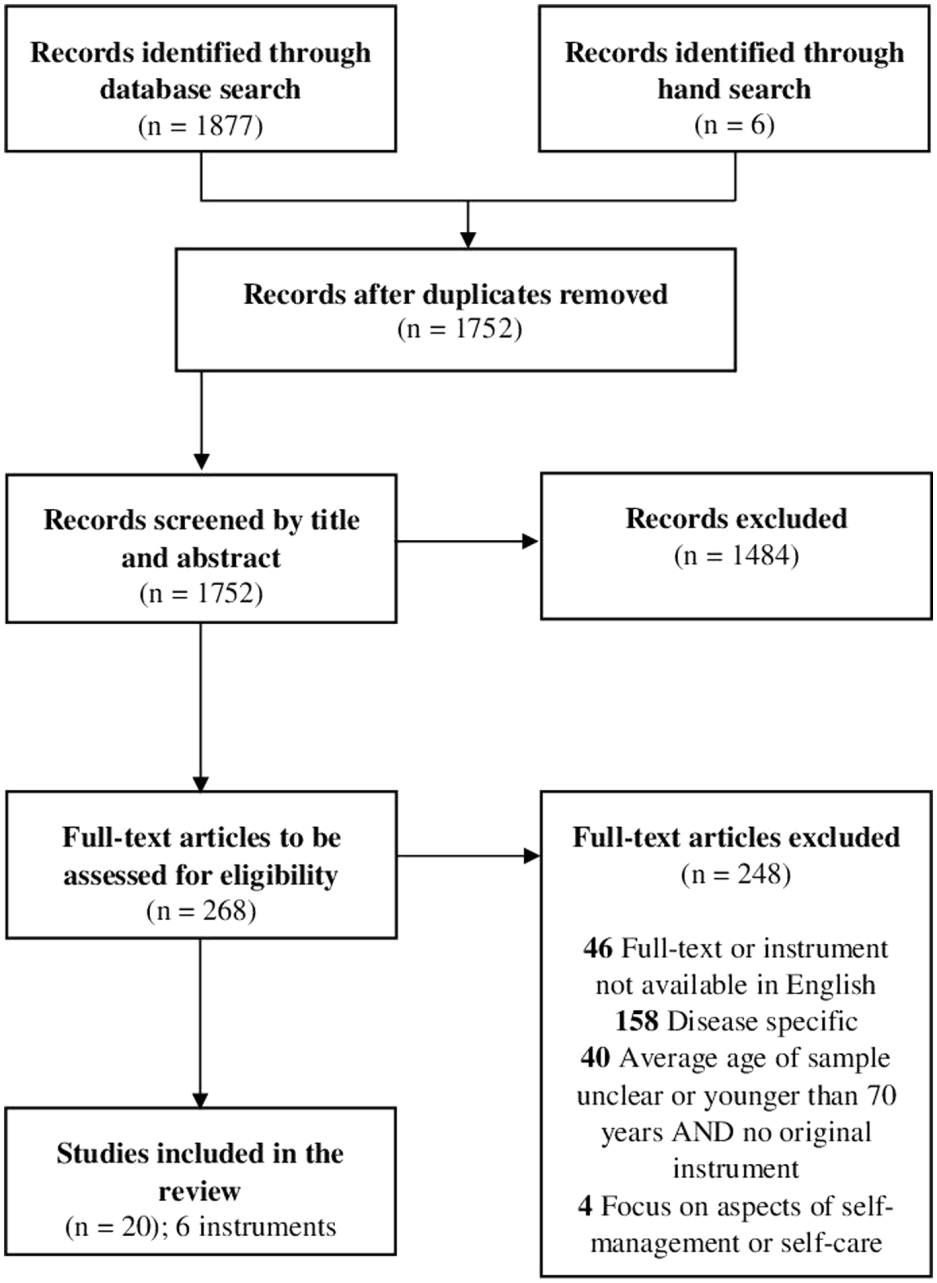
Visualization of the selection process in the style of PRISMA-ScR flow diagram.

The detailed selection process is shown in the PRISMA-ScR flow diagram (67) in Figure 1. After removal of duplicates 1752 records were identified through database searches and article references. After screening titles and abstracts, 1,484 records were excluded, resulting in the retrieval and eligibility assessment of 268 articles. Of these, 248 were excluded for the following reasons: 46 full-texts or instruments were not retrievable in English, 158 focused on instruments assessing specific diseases, 40 used a sample whose average age was unclear or less than 70 years, and were not developmental articles of considered instruments that were validated on the target population, and 4 focused on specific aspects of self-management or self-care rather than the overall concept. The remaining 20 studies were included in this review.

### 3.2 Characteristics of sources of evidence

Supplementary Table S1 provides an overview of the instruments, their definitions of self-management or self-care, the underlying theories of the instruments, and a brief sample characterization. An overview of the characteristics of the selected instruments, such as the number of items, factors or subscales, and internal consistencies, is given in Supplementary Table S2. Table 1 summarizes the item content related to the factors characterizing self-management and self-care (e.g., psychological aspects or health literacy). The different types of execution in which the item content was manifested (e.g., behavior or attitude) are shown in Table 2.

### 3.3 Results of individual sources of evidence

#### 3.3.1 The Appraisal of Self-Care-Agency-Scale (-Revised) ASAS(-R)

Orem’s Self-Care Deficit Theory (42) was the underlying premise for the development of the ASAS (68), emphasizing the importance of self-care agency. Self-care involves the actual execution of operations aimed to maintaining health, well-being, and life, whereas self-care agency refers to the ability to realize those operations required for self-care (42, 43). These operations include the investigation, decision, and performance of specific actions to meet self-care needs that refer to either basic needs, such as food intake or balancing rest and activity states, or to health- and treatment related aspects, such as symptom monitoring or medication intake. A self-care deficit occurs when individuals are unable to adequately satisfy their own needs and therefore depend on (nursing) support. Three components characterize self-care agency, namely the foundational (e.g., sensation, perception, memory, and orientation), operational (e.g., taking medication), and enabling or power components (e.g., having physical energy for self-care) (42, 43, 69, 70), with the ASAS capturing the latter (69–72). Consequently, the ASAS evaluates an individual’s ability to fulfill their overall self-care, with 24 items in the original (68, 72) and 15 items in the revised version (69). The items primarily capture actual behavior in terms of controlling diseases and adapting to new circumstances (e.g., “*If my mobility is decreased, I make the needed adjustments*”). Nine items of the ASAS and four items of the ASAS-R are negatively worded and, therefore, reverse-coded. The items of both scales can be answered by a 5-point Likert scale, ranging from 1 (totally disagree) to 5 (totally agree) (68, 69, 72). Thus, the total ASAS score ranges from 24 to 120, whereas people applying the ASAS-R can achieve a total score ranging from 15 to 75. The higher the score, the greater the self-care agency of the person. There are two versions of the ASAS: one being conducted as self-report by a person (format a) and the second assessing the self-care agency of a person through someone external (e.g., spouse or nurse; format b) (68, 72). In comparison to the unidimensional ASAS, the ASAS-R exhibits a multidimensional structure with loadings on three factors, indicating a three-factor solution better fits the data (69, 70, 73). The factors are “having capacity for self-care” (6 items), “developing capacity for self-care” (5 items), and “lacking capacity for self-care” (4 items).

#### 3.3.2 The Partners in Health Scale for older adults PIH-OA

Due to the lack of a generic self-management scale in the early 2000s, Battersby et al. (74) developed the Partners in Health Scale PIH, which is suitable for people with multiple chronic conditions rather than specific conditions. After revision, the scale consisted of twelve items (75) and revealed a four-factor structure: “knowledge” (4 items), “coping” (3 items), “management of symptoms” (3 items), and “adherence to treatment” (2 items). Response options ranged from 0 (very good/always) to 8 (very poor/never), with lower scores indicating better self-management knowledge and behavior. The item content is based on the authors’ six principles of chronic disease self-management: understanding one’s condition, adhering to a treatment plan, engaging in shared decision-making, monitoring and managing symptoms, managing the condition’s impact on life, and embracing health-promoting lifestyles (74, 75).

Because of the potential of the PIH to capture self-management not only in chronically ill adults but also in community-dwelling older adults, Veldman et al. (76) developed the PIH-OA, and validated this eight-item adaptation in a Dutch population. To make the PIH-OA suitable for older adults regardless of their health status, the term “health condition” of the PIH was changed to “consequences of aging” in the development process. Because of its focus on self-management knowledge and behaviors, the item content primarily refers to health literacy (e.g., “*In general, this is what I know about care and support for the consequences of growing older*”) and monitoring behaviors (“*I take action when my body sends me signals that I am not very well, or when I notice that the consequences of growing older are becoming more serious for me*”). The nine-point rating scale and total score (0–64) of the PIH-OA can be interpreted inversely to that of the PIH, with low scores representing poor (0 = a little/sometimes) and high scores (8 = a lot/always) representing good self-management knowledge and behaviors. Initially, the scale consisted of fourteen items, as the content of two items of the PIH was split into two new items each. To test the general acceptability of the items, the response option “not applicable” was also introduced. As six care-related items were answered with this response option by a large number of participants (both healthy and ill and in need of care), these items were deleted (e.g., “*I decide about care and supervision along with the relevant care provider*”). Factor analysis yielded three subscales: “knowledge” (2 items), “management” (2 items), and “coping” (4 items). Differences were found between the education level and health status groups, as those with higher education levels or good health status reported better self-management knowledge and behavior.

#### 3.3.3 The Patient Activation Measure (-13) PAM(-13)

The starting point of PAM development was the theory of patient activation, as outlined in the Chronic Illness Care Model (34), whose authors see activated patients as effective members of their own care team, with the skills, knowledge, and motivation to participate. Due to the lack of conceptual uniformity of patient activation, Hibbard et al. (77) developed and validated the 21 items Patient Activation Measure PAM in a comprehensive conceptualization process. The validation of a version shortened by eight items, the PAM-13, was published in 2005 (57). After an initial literature review, an expert panel, and a patient focus group, a definition emerged that specified the necessary beliefs, knowledge, skills, and behavioral repertoires that constitute an activated patient. Hence, activated patients recognize their significant role in self-managing care, collaborating with healthcare providers, and maintaining their health. They possess the knowledge and abilities to manage their condition, prevent health decline, collaborate effectively with healthcare professionals, and access high-quality care. With both the 21 items of the PAM (77) and the 13 items of the PAM-13 (57) the level of an individual’s activation can be assessed.

The item content primarily refers to confidence in doing something, especially anticipated disease-controlling behavior (e.g., “*I am confident that I can follow through on medical treatments I need to do at home*”) and knowledge in the sense of health-literacy (e.g., “*I know what each of my prescribed medications do*”). Each item has five response categories, ranging from 1 (disagree strongly) to 4 (agree strongly) and including “not applicable.” The overall score is converted into a metric from 0 to 100, with higher scores indicating higher levels of patient activation. According to the authors, patient activation is developmental and follows a hierarchical order on the activation continuum, which is executed in four stages of the activation level (77). While the first stage only involves beliefs about the importance of the patient’s role, the second includes the confidence and knowledge necessary to take action (e.g., medication and lifestyle changes, knowing when to seek help, or different treatment options). In the third stage, patients take actions (e.g., symptom management or maintaining lifestyle changes). When patients are able to stay in the course, even under stress, they reach stage four. Depending on where patients land on the activity continuum, practical implications for appropriate interventions emerge. Those scoring at the bottom of the continuum may believe that their doctors are in charge of their health. Thus, working on self-awareness of their own active part in their health process is indicated (57). In contrast, patients who land in the upper half of the continuum may feel the need to practice maintaining lifestyle changes, even in stressful, unanticipated situations. Consequently, only when the diverse needs that arise in the four stages are adequately addressed can patients become effective self-managers.

#### 3.3.4 The Self-Care Ability Scale for Elderly SASE

According to Söderhamn et al. (78) the authors of the SASE, self-care behaviors consist of three components: immediate and sustained illness-related behavioral responses, basic coping strategies, and health-maintenance actions. Pörn’s Health and Adaptedness Theory (79) served as the basis for the SASE instrument. He described humans as acting subjects whose health is determined by an equilibrium between repertoire (ability), goals, and the environment. Generalized adaptedness is the key to achieving this balance, and it occurs when humans adapt to environmental conditions and significant outcomes. Applied to self-care ability, adaptedness is defined as the capacity (intention and ability) for care of self in the three dimensions of repertoire, goals, and environment, with care including actions to maintain, improve, or prevent deterioration of well-being (78). While the repertoire comprises actions, knowledge, and decisions that encompass activities of daily living (e.g., hygiene and dressing) and instrumental activities of daily living (e.g., groceries, housekeeping), the goals represent the individual’s intention to carry out significant actions. The environmental dimension describes the human’s context (physical, psychological, and cultural). The SASE measures self-care ability among older adults using 17 items, 4 of which are negatively stated and need to be reverse-coded. The majority of the items refer to abilities and wants to execute (instrumental) activities of daily living (e.g., “*I want to manage to do my own daily shopping,” “I can in a satisfying way maintain my personal hygiene*”). Participants respond to each item using a 5-point Likert scale, ranging from 1 (totally disagree) to 5 (totally agree), with a maximum score of 85 (78). The higher the score, the higher the self-care ability. Three factors emerged from the scale, namely, capacity for care of repertoire, capacity for care of well-being, and capacity for care of goals (80), which were also confirmed in the Chinese adaptations (81, 82), but with different item compositions.

#### 3.3.5 The Self-Care of Chronic-Illness Inventory SC-CII

The Middle Range Theory (50) provided the foundation for the development of the SC-CII (83). According to this theory, self-care is practiced in both healthy and ill conditions in the form of a naturalistic decision-making process involving the preservation of health through health promotion and illness management (50, 83). Self-care is thereby executed on three dimensions: self-care maintenance (behaviors for physical and emotional stability, e.g., exercise and healthy diet), self-care monitoring (self-observation of signs and symptom changes, e.g., shortness of breath), and self-care management (responding to signs and symptoms, e.g., taking extra diuretics). The SC-CII consists of 20 items and three scales with either one- or two-factor structures according to confirmatory factor analysis: Self-Care Maintenance (factors: illness-related and health-promoting behavior), Self-Care Monitoring, and Self-Care Management (factors: autonomous and consulting behavior) (83, 84). Regarding concrete item content, the items primarily assess monitoring (e.g., “*How often or routinely do you monitor for medication side-effects?”*) and disease-controlling behaviors [e.g., “*How often or routinely do you try to avoid getting sick (e.g., flu shot, wash your hands)?*”]. Response options for different items vary; for instance, ranging from 1 (never) to 5 (always) or 1 (not likely) to 5 (very likely). Each scale must be scored separately because of the inability of symptom-free individuals to complete the Self-Care Management scale, which specifically targets symptom management. The scores on each scale are standardized using mathematical methods, resulting in a scale ranging from 0 to 100, with higher scores indicating better self-care.

#### 3.3.6 The Self-Management Ability Scale-30 SMAS-30

As posited by Schuurmans et al. (85), the methods by which individuals realize and uphold their well-being in the face of diminishing resources align with the fundamental pillars of successful self-management. This understanding of self-management, and therefore the SMAS-30, is grounded in the SSMA theory (20), which originates from the concept of social production functions (SPF theory). According to the SPF theory, humans pursue two primary objectives: physical well-being and social approval (86, 87). The SSMA theory emphasizes an individual’s reserve capacity to achieve and sustain physical and social well-being to age successfully (20). Well-being relies on two types of resources: direct resources (e.g., food, shelter, and friends), which directly impact well-being and tend to decrease with age and self-management abilities. Self-management abilities are essential for effectively handling direct resources to maintain and restore well-being. The SSMA theory identifies six core self-management abilities (SMAs): (I) ensuring the multifunctionality of resources, (II) maintaining variety in resources, (III) keeping a positive frame of mind, (IV) investing in resources for long-term benefits, (V) being self-efficacious with regard to managing resources, and (VI) taking the initiative (20). These abilities often occur simultaneously and reinforce each other (85) and are linked to the dimensions of well-being of the SPF theory, such as comfort and stimulation for physical well-being or affection (86–88). The original SMAS-30 consists of 30 Dutch items, and an English (not validated) version is provided (85). For each self-management ability, a subscale containing five items was generated, with the majority assessing behaviors concerning the social environment (e.g., “*The activities I enjoy, I do together with others*”) and lifestyle factors (e.g., *“How many hobbies or activities do you have on a regular basis?”*). The response options depend on the subscales and vary between 5-point and 6-point Likert scales. To generate an overall score, averages of each unidimensional subscale are calculated, summed, and mathematically transformed to a 100-point-scale, with a higher score indicating higher self-management abilities (18).

## 4 Discussion

This review identified six self-management or self-care instrument that have been applied to the geriatric population: ASAS-R, PAM-13, PIH-OA, SASE, SC-CII, and SMAS-30. Although all the instruments have different approaches and focus on different aspects of the two constructs, they all aim to provide insights into a person’s ability to care for themselves, make decisions related to their health and well-being, and effectively manage their resources and health conditions.

### 4.1 Underlying theories of the instruments

With the exception of the PIH(-OA), each instrument is based on a specific theoretical framework that has shaped its development and guides its interpretation. Regarding the second objective, we found out that self-management instruments are based on the Patient Activation Theory (34, 77) and the Theory of Successful Self-Management of Aging (20, 85), whereas self-care instruments are derived from Orem’s Self-Care Deficit Theory (42, 43, 68, 69, 72), the Theory of Health and Adaptedness (78, 79), and the Middle Range Theory of Self-Care and Chronic Illness (50, 83). All of these theories revolve around the idea of empowered individuals taking an active role in their care process. They emphasize the importance of individuals engaging in behaviors and actions to maintain and improve their health, such as acquiring knowledge, making decisions, and collaborating with healthcare professionals. Theories vary in their focus and understanding of the underlying components of self-care or self-management. For example, the Theory of Successful Self-Management (20, 85) of Aging identifies six specific core self-management abilities, such as ensuring the multifunctionality of resources or being self-efficacious in managing them, that are not explicitly addressed in the other theories. The Theory of Health and Adaptedness (78, 79) emphasizes the equilibrium between repertoire, goals, and environment in achieving self-care ability, whereas the Theory of Self-Care and Chronic Illness (50, 83) identifies maintenance, monitoring, and management if self-care as essential dimensions of self-care.

### 4.2 Definition of self-management and self-care

Given these different theoretical backgrounds, it is not surprising that the authors’ definitions of self-management and self-care also differ. For example, the SMAS-30 (85) is the only instrument whose definition focuses specifically on aging issues and how individuals can maintain well-being in the face of declining resources. Each author has adopted a different perspective on self-care or self-management. For example, PAM (77) emphasizes the beliefs and skills of activated individuals, whereas SC-CII (83) highlights self-care as a decision-making process, involving health-promoting practices and illness management. The context also varies. While ASAS (68) and SASE (78) address broader health and well-being, PAM (77) and SC-CII (83) focus on managing specific health aspects or concerns. All these instruments have in common, that they emphasize the importance of maintaining health and preventing health decline. They focus on promoting well-being and managing conditions effectively. Self-management and self-care are typically considered as personal responsibilities and actions taken on one’s own behalf, with the PIH(-OA) (74) authors emphasizing collaboration with carers or health professionals to empower individuals to effectively manage their health.

### 4.3 Differences and commonalities of item-content

In terms of item content as shown in Table 2, all instruments included at least one item on psychological/emotional aspects, and five out of the six instruments contained items on lifestyle factors (except PAM-13) and (anticipated) adjustment behavior (except SMAS-30). While many studies emphasize the importance of psychological well-being for successful self-management (2, 22) and self-care (42–44), it is essential to note that although all these instruments address psychological/emotional aspects at least once, they are limited in their assessment of this area. Therefore, when using these instruments, additional questionnaires on psychological well-being are recommended to gain a comprehensive understanding, such as the Geriatric Depression Scale (GDS) (89). The same applies to instrumental activities of daily living, as these are only surveyed in the SASE and with few items in the PIH-OA. Hence, if other instruments are used, it is necessary to screen them with common instruments such as the Barthel Index (90) for the activities of daily living or the Blaylock Score (91) for the instrumental activities of daily living. Health literacy, monitoring behavior, social environment, and relationships with providers are also crucial factors for successful self-management (21, 28, 31) or self-care (40, 41). However, only few instruments include them, making it necessary to either use additional questionnaires or combine existing ones to cover all relevant areas effectively, with the latter option being more economical. The PIH-OA is the most comprehensive instrument in terms of content categories, covering eight out of ten with one item each. Although ASAS-R and SC-CII each cover seven and PAM-13 covers six content categories, they each do so with more items, making them more representative of their respective concepts.

As shown in Table 2, the majority of the items in ASAS(-R), SC-CII, and SMAS-30 are related to actual behaviors, whereas PIH-OA and SASE focus on abilities, and PAM-13 primarily assesses confidence in realizing certain behaviors. Items that address feelings or wants are only used in the SASE. To better understand how geriatric patients implement daily self-management or self-care, the obstacles they might face and what needs to be addressed in tailored interventions, it is beneficial to include various types of item execution such as behavior, feelings, or attitudes in the assessment. Therefore, a combination of instruments should be considered to capture these aspects. At the same time, it is important to ensure that the items cover all relevant factors influencing self-management or self-care. Although PIH-OA contains most of the content factors, it only captures them in relation to three types of item execution: behavior, abilities and knowledge. The same is true for ASAS(-R) and SC-CII, where only two types of execution are used: behavior and attitudes for ASAS(-R) and behavior and knowledge for SC-CII. As for the SASE, its ratio of content factors and types of item execution is balanced at 5 each. By combining SASE with PAM-13, PIH-OA with PAM-13 or ASAS(-R) with PAM-13, a comprehensive collection of items that adequately covers almost all content factors sufficiently as well as a variety of types of item execution could be achieved. In the latter combinations, the (i) ADLs should be included via an additional questionnaire.

### 4.4 Limitations

A substantial number of studies were screened and processed in this review. However, it is important to note that the search may not be exhaustive, as it was restricted to articles in English. Thus, conducting further searches in various databases for articles in other languages is recommended. Furthermore, although the instruments described in detail in this review for measuring self-management or self-care in people over 70 years of age have partly been adapted in different countries, such as the SASE, their international generalizability remains uncertain. It is also worth noting that although the instruments were validated in a population with an average minimum age of 70 years, most of whom had chronic conditions, it remains unclear whether each individual met the criteria of a geriatric patient.

## 5 Conclusion

This review identified six self-management and self-care instruments suitable for geriatric patients. To the authors’ knowledge, the review thereby provides the first comprehensive overview of these two constructs pertaining to this population. Each instrument is based on a unique theory and definitional understanding of self-management and self-care, offering diverse perspectives on how individuals can actively participate in maintaining and improving their health and well-being. This heterogeneity aligns with other studies emphasizing the need for multiple instruments to assess all relevant aspects of self-management or self-care (30, 64, 92, 93). Overall, this review offers clinicians and researchers a valuable overview of appropriate instruments for assessing self-management and self-care in geriatric patients. The knowledge gained can serve as a basis for developing self-management or self-care interventions that can be tailored to the specific individual needs of geriatric patients, and as a cornerstone for testing their effectiveness.

## Data availability statement

The original contributions presented in the study are included in the article/Supplementary material, further inquiries can be directed to the corresponding author.

## Author contributions

RW: Conceptualization, Investigation, Methodology, Visualization, Writing – original draft. RB: Writing – review & editing. AS: Writing – review & editing. TP: Writing – review & editing.
