# Supplementary material for: Instruments for measuring self-management and self-care in geriatric patients – a scoping review
Source: Front Public Health. 2023 Dec 15;11:1284350. doi: 10.3389/fpubh.2023.1284350 (PMC10773718; doi:10.3389/fpubh.2023.1284350)
Supplement: Supplementary file 1 [file Data_Sheet_1.docx]

# *8Supplementary Material*

**Instruments for Measuring Self-Management and Self-Care in Geriatric Patients – a Scoping Review**

Rebecca Wientzek^1*^, Rosa Marie Brückner^1^, Aline Schönenberg^1^, Tino Prell^1,2^

***Correspondence:** Rebecca Wientzek: [rebecca.wientzek@uk-halle.de](mailto:rebecca.wientzek@uk-halle.de)

# Supplementary Figures and Tables.

Supplementary Figure. Search strategy and key search terms used in the PubMed database

Search: **(((self-management[tiab] OR self-care[tiab]) AND (measure OR assess)) AND (((questionnaire[tiab]) OR (instrument[tiab])) OR (scale[tiab]))) AND ((validation[tiab]) OR (development[tiab]))**

("self-management"[Title/Abstract] OR "self-care"[Title/Abstract]) AND ("measurability"[All Fields] OR "measurable"[All Fields] OR "measurably"[All Fields] OR "measure s"[All Fields] OR "measureable"[All Fields] OR "measured"[All Fields] OR "measurement"[All Fields] OR "measurement s"[All Fields] OR "measurements"[All Fields] OR "measurer"[All Fields] OR "measurers"[All Fields] OR "measuring"[All Fields] OR "measurings"[All Fields] OR "measurment"[All Fields] OR "measurments"[All Fields] OR "weights and measures"[MeSH Terms] OR ("weights"[All Fields] AND "measures"[All Fields]) OR "weights and measures"[All Fields] OR "measure"[All Fields] OR "measures"[All Fields] OR ("assess"[All Fields] OR "assessed"[All Fields] OR "assessement"[All Fields] OR "assesses"[All Fields] OR "assessing"[All Fields] OR "assessment"[All Fields] OR "assessment s"[All Fields] OR "assessments"[All Fields])) AND ("questionnaire"[Title/Abstract] OR "instrument"[Title/Abstract] OR "scale"[Title/Abstract]) AND ("validation"[Title/Abstract] OR "development"[Title/Abstract])

**Translations**

**measure:** "measurability"[All Fields] OR "measurable"[All Fields] OR "measurably"[All Fields] OR "measure's"[All Fields] OR "measureable"[All Fields] OR "measured"[All Fields] OR "measurement"[All Fields] OR "measurement's"[All Fields] OR "measurements"[All Fields] OR "measurer"[All Fields] OR "measurers"[All Fields] OR "measuring"[All Fields] OR "measurings"[All Fields] OR "measurment"[All Fields] OR "measurments"[All Fields] OR "weights and measures"[MeSH Terms] OR ("weights"[All Fields] AND "measures"[All Fields]) OR "weights and measures"[All Fields] OR "measure"[All Fields] OR "measures"[All Fields]

**assess:** "assess"[All Fields] OR "assessed"[All Fields] OR "assessement"[All Fields] OR "assesses"[All Fields] OR "assessing"[All Fields] OR "assessment"[All Fields] OR "assessment's"[All Fields] OR "assessments"[All Fields

Supplementary Table 1. Generic instruments measuring self-management or self-care – Definition, Background and Sample Characteristics

| **Name of instrument** | **Original instrument** | **Original or adaptation of selected validation study on people aged ≥ 70 years** | **Definition/Understanding of Self-Management/Self-Care of original instrument** | **Theoretical Background of original instrument** | **Sample Characteristics of studies validated on people aged ≥ 70 years** |
| --- | --- | --- | --- | --- | --- |
| Appraisal of Self-Care Agency Scale-Revised ASAS-R | ASAS Evers (1989) (1) Evers et al. (1993) (2)  ASAS-R Sousa et al. (2010) (3) | Chinese adaptation of ASAS-R (4)  Spanish adaptation of ASAS-R (5) | *Definition cited by Orem (1985), p.84* (6): “Self-Care is the practice of activities that individuals initiate and perform on their own behalf in maintaining life, health and well-being.” (p.8) (1) | Orem`s Self-Care Deficit Theory | Original ASAS N=140, N*female*=80 %, M*age*=80.4, Range*ag*e=65-97 N*care-dependent*=100  Chinese adaptation of ASAS-R N=1219, N*female*=49.1 %, M*age*=71.3, SD*age*=7.8, Range*ag*e=60-95 N*hospitalized*=609  Spanish adaptation of ASAS-R N=488, N*female*=62.9 %, M*age*=77.4, SD*age*=NA, Range*ag*e=65-92 |
| Patient Activation Measure-13 PAM-13 | PAM Hibbard et al. (2004) (7)  PAM-13 Hibbard et al. (2005) (8) | Swedish adaptation of PAM-13 (9) | *Definition of Patient Activation:* “Those who are activated believe patients have important roles to play in self-managing care, collaborating with providers, and maintaining their health. They know how to manage their condition and maintain functioning and prevent health declines; and they have the skills and behavioral repertoire to manage their condition, collaborate with their health providers, maintain their health functioning, and access appropriate and high-quality care.” (p.1010) (7) | Patient Activation as included in the Chronic Illness Care Model (10) | Swedish Adaptation of PAM-13 N=248, N*female*=48.4 %, Mdn*age*=70, Range*ag*e=20-96 After discharge from hospital or Virtual Health Room |

Supplementary Table 1. (Continued)

| **Name of instrument** | **Original instrument** | **Original or adaptation of selected validation study on people aged ≥ 70 years** | **Definition/Understanding of Self-Management/Self-Care of original instrument** | **Theoretical Background of original instrument** | **Sample Characteristics of studies validated on people aged ≥ 70 years** |
| --- | --- | --- | --- | --- | --- |
| Partner in Health Scale for older adults PIH-OA | PIH Battersby et al. (2003) (11)  PIH-Revised Petkov et al. (2010) (12) | Dutch adaptation of PIH-Revised for older adults: PIH-OA (13) | “Self-management involves the individual working in partnership with their carer(s) and health professionals so that (s)he can: 1. Know their condition and various treatment options. 2. Negotiate a plan of care; (i.e., Care Plan). 3. Engage in activities that protect and promote health. 4. Monitor and manage the symptoms and signs of the condition(s). 5. Manage the impact of the condition on physical functioning, emotions and interpersonal relationship” (p. 43) (11) | N/A | Dutch adaptation PIH-OA N=1127, N*female*= 55.5%, M*age*=81.7, SD*age*=4.48, Range*ag*e=75-100 N*poorhealthstatus*=17.5% |

Supplementary Table 1. (Continued)

| **Name of instrument** | **Original instrument** | **Original or adaptation of selected validation study on people aged ≥ 70 years** | **Definition/Understanding of Self-Management/Self-Care of original instrument** | **Theoretical Background of original instrument** | **Sample Characteristics of studies validated on people aged ≥ 70 years** |
| --- | --- | --- | --- | --- | --- |
| Self-Care Ability Scale for the Elderly SASE | Söderhamn et al. (1996) (14) | Chinese adaptation A (15)  Chinese adaptation B (16)  Italian adaptation (17)     Norwegian adaptation (18) | “Self-care behaviour consists of the individual’s immediate and continuing behavioural reactions to illness, the basic coping strategies and steps taken to preserve and maintain personal health.” (p. 69) (14) | Theory of Health and Adaptedness (19) | Original SASE N=57, N*female*=56.1 %, M*age*=75.6, SD*age*=5.9 N*hospitalized*=57  Chinese Adaptation A N=2856, N*female*=58.2 %, M*age*=71.5, SD*age*=7.7, Range*ag*e=60-92, N*outpatient*=2856  Chinese Adaptation B N=610, N*female*=46.4 %, M*age*=72.3, SD*age*=8.1, Range*ag*e=60-95  Italian Adaptation N=402, N*female*=60.2 %, M*age*=75.9, SD*age*=7, Range*ag*e=65-95 N*inpatient*=91, N*outpatient*=311  Norwegian Adaptation N=158, N*female*=58.2 %, M*age*=73.2, SD*age*=6.9, Range*ag*e=65-95 N*supportneeded*=23 |

Supplementary Table 1. (Continued)

| **Name of instrument** | **Original instrument** | **Original or adaptation of selected validation study on people aged ≥ 70 years** | **Definition/Understanding of Self-Management/Self-Care of original instrument** | **Theoretical Background of original instrument** | **Sample Characteristics of studies validated on people aged ≥ 70 years** |
| --- | --- | --- | --- | --- | --- |
| Self-Care of Chronic Illness Inventory SC-CII | Riegel et al. (2018) (20) | Chinese adaptation (21) | *Developed definition based on Riegel et al., (2012)* (22)*:* “[…] self-care as a naturalistic decision making process involving health-promoting practices and illness management.” (p. 2467) (20) | Middle Range Theory of Self-Care and Chronic Illness (22) | Chinese adaptation N=240, N*female*=57.5 %, M*age*=71.8, SD*age*=8.4, Range*ag*e=60-94 M*chroniccondition*=3.31, SD*chroniccondition*=1.34 |
| Self-Management Ability Scale SMAS-30 | Schuurmans et al. (2005) (23) | Original (23)  Dutch validation on recently discharged older adults (24) | “[…] the successful self-management of aging concerns the ways in which people are able to realize well-being and in particular, how they can sustain it, even when their resources decline.” (p. 2216) (23) | The Theory of Successful Self-Management of Aging SSMA (25) | Original Study 1 N=275, N*female*=72.4 %, M*age*=78.4, SD*age*=7.05, Range*ag*e=64-99 N*residents*=17.2%  Original Study 2 N=1338,  Nfemale=59 %, M*age*=74.2, SD*age*=6.59, Range*age*=65-98 N*residents*=1%  Discharged patients Study N=296, N*female*=54,2 %, Median*age*=75.8, SD*age*=6.8, Range*age*=65-94 N*residents*=6.8% |

Supplementary Table 2. Characteristics of instruments (original or adaptation) that were validated on people aged $\geq$ 70 years.

| **Name of Instrument** | **Original or adaptation of selected validation study on people aged ≥ 70 years** | **Amount of Items** | **Rating Scale** | **Identified Factors and subscales** | **Internal Consistency for instruments and subscales** |
| --- | --- | --- | --- | --- | --- |
| Appraisal of Self-Care Agency Scale Revised  ASAS-R | Chinese adaptation (4) | 15 | 5-point Likert scale (1=totally disagree, 5=totally agree) | 3: Having power for self-care Developing power for self-care Lacking power for self-care | αTotal=.79 |
|  | Spanish adaptation (5) | 15 |  | 3: Having capacity for self-care Developing capacity for self-care Lacking capacity for self-care | CRHavingCapacity=.94 CRDevelopingCapacity=.93 CRLackingCapacity=.98 |
| Patient Activation Measure-13 PAM-13 | Swedish adaptation (9) | 13 | 4-point Likert scale (1=strongly disagree, 4=agree strongly) and option “not applicable” | N/A | αTotal=.81 |
| Partner in Health Scale for older adults  PIH-OA | Dutch adaptation of PIH-Revised (13) | 8 | 9-point Likert scale (0=a little/sometimes, 8=a lot/always) and option “not applicable” | 3: Knowledge, Management, Coping | αTotal=.77 αKnowledge=.84 αManagement=.77 αCoping=.83 |
| Self-Care Ability Scale for the Elderly  SASE | Chinese adaptation A (15) | 17 | 5-point Likert scale (1=totally disagree, 5=totally agree) | 3: Repertoire, Environment, Goal | N/A |
|  | Chinese adaptation B (16) | 17 |  |  | αTotal=.89 |
|  | Italian adaptation (17) | 13 |  | 3: Self-care ability to perform ADLs, Self-care ability to achieve well-being, Self-care ability to set personal goals | αADL=.90 αwell-being=.87 αgoals=.72 |
|  | Norwegian adaptation (18) | 17 |  | N/A | αTotal=.85 |

Supplementary Table 2. (Continued)

| **Name of Instrument** | **Original or adaptation of selected validation study on people aged ≥ 70 years** | **Amount of Items** | **Rating Scale** | **Identified Factors and subscales** | **Internal Consistency for instruments and subscales** |
| --- | --- | --- | --- | --- | --- |
| Self-Care of Chronic Illness Inventory  SC-CII | Chinese adaptation (21) | 20 | 5-point Likert scale (1=never, 5=always) | 3: Self-care maintenance scale Self-care monitoring scale Self-care management scale | Ωmaintenance=.81 Ωmonitoring=.82 Ωmanagement=.77 |
| Self-Management Ability Scale SMAS-30 | Original (23) | 30 | Subscale Multifunctionality: 5-point Likert scale (1=strongly disagree, 5=strongly agree)  Subscale Variety: 6-point Likert scale (1=none, 6=more than six)  Subscale Positive Frame of Mind, Investment Behavior and Taking Initiatives: 6-point Likert scale (1=never, 6=very often)  Subscale Self-Efficacy: 5-point Likert scale (1=I’m certain that I can not, 5 = I’m certain that I can) | 6: Multifunctionality Variety Positive Frame of Mind Investment Behavior Self-efficacy Taking Initiatives | Study 1/Study 2: αTotal=.91/.91 αMultifunctionality=.71/.74 αVariety=.67/.72 αFrameofMind=.83/.84 αInvestment=.75/.72 αSelfefficacy=.82/.73 αInitiatives=.72/.75 |
|  | Dutch validation on recently discharged older adults (24) | 18 |  |  | αMultifunctionality=.69 αVariety=.69 αFrameofMind=.74 αInvestment=.71 αSelfefficacy=.77 αInitiatives=.77 |

**References**

1. Evers GCM. Appraisal of self-care agency A.S.A.-scale : reliability and validity testing of the Dutch version of the A.S.A.-scale measuring Orem’s concept “Self-Care Agency” [Internet]. maastricht university; 1989 [cited 2023 May 8]. Available from: https://cris.maastrichtuniversity.nl/en/publications/041ad0f8-3894-4e03-aee7-b8909a2e3355

2. Evers GCM, Isenberg MA, Philipsen H, Senten M, Brouns G. Validity testing of the Dutch translation of the appraisal of the self-care agency A.S.A.-scale. International Journal of Nursing Studies. 1993 Aug 1;30(4):331–42.

3. Sousa VD, Zauszniewski JA, Bergquist-Beringer S, Musil CM, Neese JB, Jaber AF. Reliability, validity and factor structure of the Appraisal of Self-Care Agency Scale – Revised (ASAS-R). Journal of Evaluation in Clinical Practice. 2010;16(6):1031–40.

4. Guo L, Zauszniewski JA, Ding X, Zhang L, Gao H, Guo Q, et al. The Appraisal of Self-Care Agency Scale-Revised (ASAS-R): Reliability and Validity Among Older Chinese People. West J Nurs Res. 2017 Nov;39(11):1459–76.

5. Alhambra-Borrás T, Durá-Ferrandis E, Garcés-Ferrer J, Sánchez-García J. The Appraisal of Self-Care Agency Scale - Revised (ASA-R): Adaptation and Validation in a Sample of Spanish Older Adults. Span J Psychol. 2017;20:E48.

6. Orem DE. Nursing: concepts of practice. 3rd ed. New York: McGraw-Hill; 1985. 303 p.

7. Hibbard JH, Stockard J, Mahoney ER, Tusler M. Development of the Patient Activation Measure (PAM): conceptualizing and measuring activation in patients and consumers. Health Serv Res. 2004 Aug;39(4 Pt 1):1005–26.

8. Hibbard JH, Mahoney ER, Stockard J, Tusler M. Development and Testing of a Short Form of the Patient Activation Measure. Health Services Research. 2005;40(6p1):1918–30.

9. Hellström A, Kassaye Tessma M, Flink M, Dahlgren A, Schildmeijer K, Ekstedt M. Validation of the patient activation measure in patients at discharge from hospitals and at distance from hospital care in Sweden. BMC Public Health. 2019 Dec 19;19(1):1701.

10. Bodenheimer T, Lorig K, Holman H, Grumbach K. Patient Self-management of Chronic Disease in Primary Care. JAMA. 2002 Nov 20;288(19):2469–75.

11. Battersby MW, Ask A, Reece MM, Markwick MJ, Collins JP. The Partners in Health scale: The development and psychometric properties of a generic assessment scale for chronic condition self-management. Aust J Prim Health. 2003;9(3):41–52.

12. Petkov J, Harvey P, Battersby M. The internal consistency and construct validity of the partners in health scale: validation of a patient rated chronic condition self-management measure. Qual Life Res. 2010 Sep 1;19(7):1079–85.

13. Veldman K, Reijneveld SA, Lahr MMH, Uittenbroek RJ, Wynia K. The Partners in Health scale for older adults: design and examination of its psychometric properties in a Dutch population of older adults. Health Expectations. 2017;20(4):601–7.

14. Söderhamn O, Ek AC, Pürn I. The Self-care Ability Scale for the Elderly. Scandinavian Journal of Occupational Therapy. 1996 Jan 1;3(2):69–78.

15. Guo L, Wei M, Namassevayam G, Söderhamn U, Liu Y, Guo Y. Factor analysis of the Chinese version of the Self-care Ability Scale for the Elderly: A multi-centre cross-cultural validation study. International Journal of Nursing Practice. 2023;29(2):e13060.

16. Gao H, Söderhamn U, Cliffordson C, Guo L, Guo Q, Liu K. Reliability and validity of the Chinese version of the Self-care Ability Scale for the Elderly. Journal of Clinical Nursing. 2017;26(23–24):4489–97.

17. Raffaele B, Biagioli V, Cirillo L, De Marinis MG, Matarese M. Cross-validation of the Self-care Ability Scale for Elderly (SASE) in a sample of Italian older adults. Scandinavian Journal of Caring Sciences. 2018;32(4):1398–408.

18. Tomstad ST, Söderhamn U, Espnes GA, Söderhamn O. Testing two self-care-related instruments among older home-dwelling people in Norway. International Journal of Older People Nursing. 2013;8(3):189–98.

19. Pörn I. Health and adaptedness. Theor Med Bioeth. 1993 Dec 1;14(4):295–303.

20. Riegel B, Barbaranelli C, Sethares KA, Daus M, Moser DK, Miller JL, et al. Development and initial testing of the self-care of chronic illness inventory. Journal of Advanced Nursing. 2018;74(10):2465–76.

21. Jin Y, Brown R, Bhattarai M, Kuo W chin, Chen Y. Psychometric properties of the self-care of chronic illness inventory in Chinese older adults with multiple chronic conditions. International Journal of Older People Nursing. 2023 Mar 14;n/a(n/a):e12536.

22. Riegel B, Jaarsma T, Strömberg A. A Middle-Range Theory of Self-Care of Chronic Illness. Advances in Nursing Science. 2012 Jul;35(3):194–204.

23. Schuurmans H, Steverink N, Frieswijk N, Buunk BP, Slaets JPJ, Lindenberg S. How to Measure Self-management Abilities in Older People by Self-report. The Development of the SMAS-30. Qual Life Res. 2005 Dec 1;14(10):2215–28.

24. Cramm JM, Strating MM, de Vreede PL, Steverink N, Nieboer AP. Validation of the self-management ability scale (SMAS) and development and validation of a shorter scale (SMAS-S) among older patients shortly after hospitalisation. Health and Quality of Life Outcomes. 2012 Jan 24;10(1):9.

25. Steverink N, Lindenberg S, Slaets JPJ. How to understand and improve older people’s self-management of wellbeing. Eur J Ageing. 2005 Dec 1;2(4):235–44.
